# Impact of regional heterogeneity of RSV infection prophylaxis on bronchiolitis in Italy

**DOI:** 10.1186/s13052-026-02211-y

**Published:** 2026-06-13

**Authors:** Raffaele Badolato, Viola Ceconi, Roberto Bellu’, Eugenio Baraldi, Chiara Azzari, Renato Cutrera, Fabio Midulla, Roberto Bernardini, Domenico Cipolla, Daniela Concolino, Alessandro Consolaro, Elisabetta Cortis, Antonio Cualbu, Marisa D’Andrea, Grazia Dinnella, Simonetta Fain, Enrico Felici, Santina Gaggiano, Paola Giordano, Alfredo Guarino, Lorenzo Iughetti, Eustachio Lapacciana, Angelo Pietrobelli, Guido Pennoni, Ermanno Ruffini, Camilla Gizzi, Massimo Agosti, Rino Agostiniani, ITARSV-Task-force

**Affiliations:** 1 Clinica Pediatrica dell’Università degli Studi di Brescia, ASST Spedali Civili, P.le Spedali civili 1, Brescia, Italy; 2 Pediatric Unit, ASST Spedali Civili, Brescia, Italy; 3 Neonatal Intensive Care Unit, Woman and Child Health Department, Azienda Ospedaliera della Provincia di Lecco, Lecco, Italy; 4 Department of Women’s and Children’s Health, University Hospital of Padova, Padua, Italy; 5 Institute of Pediatric Research, “Città della Speranza”, Padua, Italy; 6 Immunology Unit, Department of Pediatrics, Meyer Children’s Hospital IRCCS, Florence, Italy; 7 Pneumology and Cystic Fibrosis Unit, Bambino Gesù’ Children’s Hospital, IRCCS, Rome, Italy; 8 Department of Maternal Infantile and Urological Sciences, Sapienza University of Rome, Rome, Italy; 9 Head Pediatric and Neonalogy Unit, San Giuseppe Hospital, Maternal and Child Health Department, Azienda USL Toscana Centro, Empoli, Italy; 10 Neonatal Intensive Care Unit, University Hospital of Catania (G. Rodolico), Catania, Italy; 11 Pediatric Unit, Department of Health Sciences, University of Catanzaro “Magna Graecia”, Catanzaro, Italy; 12 Pediatric Rheumatology Unit, IRCCS Istituto Giannina Gaslini”, Genoa, Italy; 13 Department of Neurosciences, Rehabilitation, Ophthalmology, Genetics and Maternal-Infantile Sciences (DiNOGMI), University of Genoa, Genoa, Italy; 14 Department of Pediatrics, Sant’ Eugenio Hospital, Rome, Italy; 15 Pediatric Unit, San Francesco Hospital, Nuoro, Italy; 16 Family pediatrician, L’Aquila, Italy; 17 Pediatric Clinic, ASUIT Integrated University Health Authority of Trentino, Rovereto, Italy; 18 Family Pediatrician, ASUGI Azienda Sanitaria Universitaria Giuliano Isontina, Gorizia, Italy; 19 Pediatric and Pediatric Emergency Unit, Children Hospital, University Hospital SS Antonio e Biagio e C. Arrigo, Alessandria, Italy; 20 Pediatrics and Neonatology Units, ASReM, Molise, Italy; 21 Pediatric Clinic, Dipartiment Interdisciplinare of Medicin, University of Bari, Bari, Italy; 22 Department of Translational Medical Sciences, Section of Pediatrics, University of Naples Federico II, Via Pansini 5, Naples, Italy; 23 Pediatric Unit, Mother Children Dept, University Hospital, Modena, Italy; 24 Family Pediatrician, Department of Primary Care ASM Basilicata and Presidente Sezione Sip Basilicata, Matera, Italy; 25 Dipartimento di Scienze Chirurgiche, Odontostomatologiche e Materno Infantili, AOUI Verona, Università degli Studi di Verona, Verona, Italy; 26 Pennington Biomedical Research Center, Baton Rouge, LA, USA; 27 Direttore Dipartimento Materno-Infantile, USL Umbria1 e UOC Pediatria Città di Castello e Gubbio-Gualdo Tadino, Gualdo Tadino, Italy; 28 Pediatric and Neonatology Unit, AST 5 Hospital, Ascoli Piceno, Italy; 29 Neonatology and NICU “Sant’Eugenio” Hospital, ASL RM2, Piazzale dell’Umanesimo 10, Rome, Italy; 30 Research Centre for Nutrition, University of Insubria, Varese, Italy; 31 Maternal and Child Department, Hospital “F. Del Ponte”, University of Insubria, Varese, Italy; 32 Department of Pediatrics and Neonatology, San Jacopo Hospital, Via Ciliegiole 97, Pistoia, 51100 Italy

## Abstract

Respiratory syncytial virus (RSV) remains a leading cause of bronchiolitis and hospitalization in infants across Italy, particularly during predictable seasonal outbreaks. With the 2023–2025 RSV seasons marking the first large-scale rollout of the monoclonal antibody nirsevimab, this study aimed to evaluate the implementation strategies, regional variability, and early clinical impact of RSV prophylaxis at the national level. We conducted a nationwide, multicenter, observational study across 19 Italian regions. Data were collected through the Italian Society of Pediatrics and the Italian Society of Neonatology, focusing on regional differences in rollout timing, organizational models, logistical challenges, and RSV-related hospitalizations and pediatric intensive care unit (PICU) admissions. The results highlighted significant regional heterogeneity. Northern and central regions such as Veneto, Lombardy, and Tuscany initiated prophylaxis earlier and experienced fewer logistical barriers, resulting in marked reductions in RSV hospitalizations - up to 83.7% in Friuli-Venezia Giulia. Conversely, southern and smaller central regions, including Molise, Marche, and Umbria, faced delayed starts, supply shortages, and bureaucratic challenges, leading to more modest decreases. PICU admissions mirrored these trends. Overall, the national introduction of nirsevimab correlated with a significant reduction in RSV-related morbidity, especially in regions with early, well-organized rollouts. Comparative international studies from France, Spain, and Luxembourg reinforce the Italian findings: timely and universal prophylaxis leads to substantial public health benefits. The study concludes that early campaign activation, consistent drug availability, and efficient organization are critical for maximizing the protective effect of RSV immunization.

## Background

Respiratory syncytial virus (RSV) is a major cause of lower respiratory tract infections in infants and the leading cause of hospitalization of children in Italy in the first year of life [1]. Bronchiolitis is the most common severe manifestation, but RSV infection is associated with recurrent wheezing in infancy, and possibly with an increased risk of developing asthma [2–4]. Because of the lack of effective treatment for RSV bronchiolitis, the reduction of morbidity must rely on preventive measures, and its therapy includes general supportive management to control pulmonary and systemic clinical symptoms [5].

In Italy, RSV shows clear seasonality, but the timing and intensity vary by region. Nationwide surveillance shows RSV epidemics generally occur from late autumn to early spring, peaking between December and February, with earlier peaks in northern regions and slightly later peaks in the south. In northern and central Italy, RSV activity usually starts in November, peaks in December–January, and ends by March, while in southern Italy, the season can extend into April. A multi-season analysis confirmed that RSV circulation is highly consistent, with yearly epidemics showing limited variability in onset and peak, making seasonally targeted prophylaxis feasible [6].

In past years, the most effective RSV prevention strategies were hygiene measures and passive immunization with palivizumab for high-risk infants, while vaccines remained experimental until very recently. Early vaccine trials in the 1960s failed due to enhanced disease, but later studies showed subunit vaccines could reduce RSV incidence, though they never reached wide use [7]. More recently, maternal vaccination and new platforms have been under development [8]. RSV immune globulin (RSV-IGIV) was one of the first agents shown to reduce the incidence and severity of RSV, but its use declined due to logistical issues [9]. In particular, Palivizumab (monoclonal antibody) became the standard prophylaxis for high-risk infants (premature, congenital heart disease, bronchopulmonary dysplasia), reducing RSV hospitalizations significantly [10, 11].

In recent years a new generation of long-acting anti-RSV mAb has been developed. Nirsevimab is a monoclonal antibody designed to protect infants from RSV, and research shows it is effective in reducing severe cases of bronchiolitis caused by RSV. Nirsevimab significantly reduced RSV-related hospitalizations and severe cases of bronchiolitis in healthy infants during their first RSV season [12]. A pooled analysis found nirsevimab lowered the incidence of medically attended RSV lower respiratory tract infections and hospitalization compared to placebo, showing broad protective effects [13]. Real-world trials showed that even a single intramuscular dose provided protection across an entire RSV season, helping reduce the burden of bronchiolitis in both preterm and full-term infants [14–16]. In Valle d’Aosta a recent Study demonstrated that universal prophylaxis with nirsevimab significantly reduced RSV-related hospitalizations in neonates, with no hospitalizations in the treated group compared to 8.3% in the untreated group [17]. In Tuscany, a real-world cost-benefit analysis assessed various nirsevimab prophylaxis strategies, finding that universal.

prophylaxis targeting all infants during their first RSV epidemic season substantially reduced hospitalization burdens without increasing economic pressure on the healthcare system [18].

In order to evaluate the implementation strategies, regional variability, and early clinical impact of RSV prophylaxis at the national level, we conducted a nationwide, multicenter, observational study across 19 Italian regions by analyzing RSV-related hospitalizations and pediatric intensive care unit (PICU) admissions.

## Methods

This descriptive retrospective study was conducted in 19 Italian regions through a collaboration between the Italian Society of Pediatrics (SIP) and the Italian Society of Neonatology (SIN). The 19 regional branches of SIP collected information from their respective regions regarding the implementation methods of the RSV infection prophylaxis campaign. For each region, we reported the start date of the prophylaxis campaign during the epidemic season 2024–2025, children eligible for immunization (born during the epidemic season from October 2024 to March 2025 or ot-of-season born), major issues encountered during this first campaign.

In each region, one or more sentinel hospitals were identified: Giuseppe Mazzini hospital (Teramo, Abruzzo), Giovanni XXIII (Bari, Apulia), San Carlo hospital (Potenza, Basilicata), Annunziata hospital (Cosenza, Calabria), Santobono Pausilipon hospital (Napoli, Campania) and all the neonatal intensive care units of Campania, AUSL Bologna (Bologna, Emilia-Romagna), S. Orsola-Malpighi (Bologna, Emilia-Romagna), AUSL Modena (Modena, Emilia-Romagna), Ramazzini Hospital (Carpi , Emilia-Romagna), Guglielmo da Saliceto hospital (Piacenza, Emilia-Romagna), Santa Maria Hospital (Reggio Emilia, Emilia-Romagna), Infermi Hospital (Rimini, Emilia-Romagna), Santa Maria della Misericordia hospital (Udine, Friuli-Venezia Giulia), Bambino Gesù hospital (Roma, Lazio), Sant’Andrea hospital (Roma, Lazio), Sant’Eugenio hospital (Roma, Lazio), Umberto I hospital (Roma, Lazio), Giannina Gaslini hospital (Genova, Liguria), Bolognini hospital (Bergamo, Lombardy), Desenzano del Garda hospital (Brescia, Lombardy), Esine hospital (Brescia, Lombardy), Spedali Civili hospital (Brescia, Lombardy), Ca’ Granda hospital (Milano, Lombardy), Fatebenefratelli Sacco hospital (Milano, Lombardy), Giuseppe Fornaroli hospital (Milano, Lombardy), Legnano hospital (Milano, Lombardy), San Paolo hospital (Milano, Lombardy), San Raffaele hospital (Milano, Lombardy), Vizzolo Predabissi hospital (Milano, Lombardy), San Gerardo dei Tintori hospital (Monza, Lombardy), San Matteo hospital (Pavia, Lombardy), Galmarini-Tradate hospital (Varese, Lombardy), Salesi Ancona hospital (Ancona, Marche), Carlo Urbani hospital (Jesi, Marche), Principe di Piemonte hospital (Senigallia, Marche), Madonna del Soccorso hospital (San Benedetto del Tronto, Marche), Mazzoni hospital (Ascoli Piceno, Marche), Augusto Murri hospital (Fermo, Marche), Civitanova Marche hospital (Civitanova Marche, Marche), Macerata hospital (Macerata, Marche), San Salvatore hospital (Pesaro, Marche), Santa Croce hospital (Fano, Marche), Santa Maria della Misericordia hospital (Urbino, Marche), Antonio Cardarelli hospital (Campobasso, Molise), San Timoteo hospital (Campobasso, Molise), Ferdinando Veneziale hospital (Isernia, Molise), Cesare Arrigo hospital (Alessandria, Piedmont), Maggiore hospital (Novara, Piedmont), Giuseppe Brotzu hospital (Cagliari, Sardinia), San Francesco hospital (Nuoro, Sardinia), San Martino hospital (Oristano, Sardinia), Santissima Annunziata hospital (Sassari, Sardinia), Giovanni di Cristina hospital (Palermo, Sicily), Meyer hospital (Firenze, Tuscany), Mugello hospital (Firenze, Tuscany), San Giuseppe hospital (Firenze, Tuscany), Santa Maria Annunziata hospital (Firenze, Tuscany), Cecina hospital (Livorno, Tuscany), San Luca hospital (Lucca, Tuscany), Versilia hospital (Lucca, Tuscany), Apuane hospital (Massa-Carrara, Tuscany), Santa Chiara hospital (Pisa, Tuscany), San Jacopo Hospital (Pistoia, Tuscany), Bolzano Central hospital (Bolzano, Trentino-Alto Adige), Bressanone hospital (Bolzano, Trentino-Alto Adige), Rovereto hospital (Trento, Trentino-Alto Adige), Città di Castello hospital (Perugia, Umbria), Gubbio e Gualdo Tadino hospital (Perugia, Umbria), San Giovanni Battista (Perugia, Umbria), Santa Maria della Misericordia hospital (Perugia, Umbria), Santa Maria della Stella hospital (Terni, Umbria), Santa Maria hospital (Terni, Umbria), all the hospitals of Veneto.

For each region, we collected aggregated data from the identified sentinel hospitals on hospitalizations for RSV bronchiolitis in children aged < 2 years. Eligible cases included admissions to neonatology units, pediatric wards, and pediatric intensive care units (PICUs) occurring during the RSV epidemic periods from October to March of the 2023–2024 and 2024–2025 seasons. Specifically, we recorded: (i) the number of hospital admissions for RSV bronchiolitis in neonatology and pediatric wards, reported month by month from October 2023 to March 2024 and from October 2024 to March 2025, obtained through the sum of admissions across sentinel hospitals within each region; (ii) the cumulative number of RSV bronchiolitis–related admissions to PICUs over the same periods, calculated as the total number of PICU admissions across regional sentinel hospitals; (iii) the percentage reduction in RSV bronchiolitis–related hospital admissions in the 2024–2025 epidemic season compared with the 2023–2024 season; (iv) the percentage reduction in RSV bronchiolitis-related PICU admissions across regional sentinel hospital. RSV bronchiolitis cases were identified using the hospital discharge coding systems routinely adopted in each center for RSV case classification. Hospitalizations in children aged ≥ 2 years, admissions outside the predefined epidemic periods, and cases lacking a clear RSV bronchiolitis diagnostic code were excluded. Data were collected and analyzed in aggregated form only, without access to individual-level patient information.

Because data were derived from selected sentinel hospitals, the reported hospitalization figures may not fully represent the overall regional burden of RSV bronchiolitis, and differences in hospital catchment areas and referral patterns should be considered when interpreting regional comparisons.

### Statistical analysis and calculation of outcomes

Regional data on nirsevimab implementation policies, RSV-related hospitalizations, and pediatric intensive care unit (PICU) admissions were collected at an aggregate level from 19 Italian regions through the Italian Society of Pediatrics and the Italian Society of Neonatology. Reductions in RSV-related hospitalizations and PICU admissions were calculated for each region by comparing the absolute number of reported cases in the 2024–2025 season with those reported in the 2023–2024 season. The percentage change was computed as the relative reduction between seasons, using the previous season as the reference. These comparisons were intended to describe temporal trends within regions rather than to estimate causal effects.

Analyses were primarily descriptive. Regional outcomes were compared qualitatively and quantitatively to highlight patterns of variability in implementation timing, organizational capacity, and observed changes in disease burden. Regions were subsequently grouped into three performance categories based on a combination of policy implementation characteristics (timing of rollout, logistical barriers, drug availability) and the magnitude of observed reductions in hospital admissions. These groupings were exploratory and designed to facilitate interpretation of heterogeneous regional experiences rather than to represent statistically derived clusters. Formal inferential statistical testing was not performed. This decision was based on several considerations: the observational design of the study; the use of aggregated, region-level data rather than individual-level records; the absence of standardized denominators (such as precise regional immunization coverage rates or age-stratified population at risk); and potential variability in case ascertainment and reporting practices across regions. Under these conditions, inferential testing could have produced misleading estimates or implied causal relationships not supported by the data structure. Accordingly, results are presented as descriptive comparisons aimed at illustrating real-world variability in implementation strategies and associated outcomes. Findings should therefore be interpreted as indicative of trends and associations rather than definitive measures of effectiveness.

## Results

We have collected the vaccination and immunization recommendations from 19 Italian regions and analyzed differences and outcomes. The rollout of nirsevimab prophylaxis against RSV in Italy has been marked by wide heterogeneity among regions, with differences in start dates, organizational barriers, and drug availability. Some regions were able to launch their campaigns early and without significant obstacles, while others faced delays, shortages, and bureaucratic challenges that slowed implementation (Table 1).

**Table 1:** Heterogeneity of nirsevimab prophylaxis among Italian regions

Region	Campaign start date	Issues
Abruzzo	07-Jan-25	Delayed activation, tender process.
Apulia	12-Nov-24	Short periods of drug unavailability.
Basilicata	06-Dec-24	Not specified.
Calabria	16-Dec-24	Difficult coverage of previously born infants, low-dose availability.
Campania	11-Nov-24	Bureaucratic difficulties in entering newborns into the platform.
Emilia-Romagna	28-Oct-24	None.
Friuli-Venezia Giulia	04-Nov-24	Initial difficulties in obtaining doses, then regular start.
Lazio	11-Dec-24	Late start and limited dose availability.
Liguria	05-Dec-24	Drug availability/number of doses.
Lombardy	01-Nov-24	None.
Marche	01-Dec-24	Delayed drug availability.
Molise	20-Nov-24	Drug unavailable from the second half of December 2024.
Piedmont	01-Nov-24	None.
Sardinia	16-Dec-24	Lack of availability of 100 mg vials.
Sicily	01-Nov-24	Not specified.
Tuscany	04-Nov-24	None.
Trentino-Alto Adige	28-Oct-24	None.
Umbria	20-Jan-25	Regional Council resolution at the end of November 2024. Delay in doses.
Veneto	04-Nov-24	None.

In Northern and Central Italy, several regions demonstrated strong preparedness. Emilia-Romagna and Trentino-Alto Adige were among the first to start at the end of October 2024, followed closely by Lombardy, Piedmont, Tuscany, and Veneto in early November. These regions largely reported no major issues, indicating effective planning and coordination. Friuli-Venezia Giulia also began in early November, experiencing only brief difficulties in obtaining doses before establishing regular supply.

By contrast, other regions encountered interruptions or barriers that slowed the campaign. Apulia, starting in mid-November, faced short periods of unavailability. Marche launched at the beginning of December but was affected by the delayed delivery of doses. Liguria and Lazio, both beginning in December, struggled with limited availability, while Campania dealt with bureaucratic obstacles in enrolling newborns into the digital platform. Calabria and Sardinia, also starting in December, reported more severe problems, including difficulties in covering infants born before the campaign began, insufficient doses, and lack of 100 mg vials. Molise, despite starting in late November, experienced a complete absence of supply from January onward.

Other regions began later still. Basilicata activated prophylaxis in December but did not provide specific details on difficulties. Abruzzo delayed its start until early January 2025 due to tender procedures, and Umbria only launched in late January, slowed by a late Regional Council resolution and limited drug availability.

## Report on RSV admissions in Italian hospitals (2023–2024 vs. 2024–2025 seasons)

Hospital admissions of neonates and infants with RSV infections during the last two seasons show a marked overall decline across Italian regions, although the extent of reduction varied significantly from North to South. The data indicate both widespread decreases in case burden and striking regional differences in the magnitude of reduction (Table 2).

**Table 2:** Percentage decreases in neonatology and pediatrics admissions and in PICU admissions among Italian regions

Region	Neonatology and pediatrics admissions			PICU admissions		
	2023–2024	2024–2025	Changes	2023–2024	2024–2025	Changes
Abruzzo	79	47	-40.5%	4	4	-0%
Apulia	118	58	-50.8%	nd	nd	-
Basilicata	83	53	-36.1%	11	3	-73%
Calabria	32	9	-71.8%	3	0	-100%
Campania	555	336	-39.4%	41	23	-44%
Emilia-Romagna	519	305	-41.2%	41	19	-54%
Friuli-Venezia Giulia	98	16	-83.7%	61	6	-90%
Lazio	438	294	-32.9%	59	17	-72%
Liguria	227	120	-47.1%	nd	nd	-
Lombardy	832	300	-63.9%	127	38	-70%
Marche	210	179	-14.8%	10	2	-80%
Molise	48	37	-22.9%	0	0	-
Piedmont	148	53	-64.2%	13	7	-46%
Sardinia	158	75	-52.3%	9	2	-78%
Sicily	1529	822	-46.2%	93	25	-73%
Tuscany	483	118	-75.6%	66	12	-82%
Trentino-Alto Adige	154	91	-40.9%	14	3	-79%
Umbria	179	146	-18.4%	25	12	-52%
Veneto	1203	379	-68.5%	116	27	-77%

Several regions recorded very steep declines. Friuli-Venezia Giulia experienced the most dramatic drop, from 98 admissions in 2023–2024 to only 16 in 2024–2025, corresponding to nearly 84% fewer cases. Lombardy, one of the most populous regions, saw a substantial reduction of nearly two-thirds, moving from over 800 cases to 300. Piedmont, Tuscany and Veneto also showed pronounced declines, with reductions above 64%, 76% and 68% respectively.

Regions in the South also reflected meaningful decreases, though with different intensities. Sicily, which had by far the highest burden in the previous season with more than 1,500 admissions, reported a 46% decline but still accounted for over 800 cases in 2024–2025. Campania and Apulia also showed decreases of around 40–50%, while Calabria achieved a more dramatic reduction of almost 72%, moving from 32 cases down to only 9. But, this is the result of analysis a single hospital from the region. Sardinia’s admissions dropped by over half, confirming a significant downward shift.

In Central Italy, Lazio recorded nearly a 33% decrease, falling from 438 admissions to 294. Marche saw only a modest reduction of about 15%, with 179 cases still reported. Umbria and Molise also showed more contained decreases, of 18% and 23% respectively, suggesting that RSV activity remained relatively stable compared to the stronger declines observed elsewhere.

Regions of the North generally saw consistent reductions. Emilia Romagna, with over 500 cases in 2023–2024, reported a 41% decline to 305 admissions. Liguria recorded a nearly 47% decrease. TrentinoAlto Adige showed a 41% reduction, while Basilicata, despite lower absolute numbers, saw a significant decline of 36%.

## Report on PICU admissions for RSV in Italian regions (2023–2024 vs. 2024–2025 seasons)

The number of admissions to Pediatric Intensive Care Units (PICU) for RSV infection decreased substantially across most Italian regions between the 2023–2024 and 2024–2025 seasons, though with notable variability in magnitude and distribution (Table 2).

Regions with the highest case burden in the previous season showed marked reductions. Lombardy dropped from 127 admissions to 38, representing a 70% decrease, while Sicily, with 93 admissions previously, recorded a 73% reduction to 25. Friuli Venezia Giulia showed one of the most dramatic improvements, falling from 61 to just 6 cases, equivalent to a 90% decrease. Emilia Romagna also demonstrated strong progress, cutting admissions by more than half, from 41 to 19. Campania mirrored this trend, moving from 41 to 23 admissions, a 44% reduction.

Other regions with moderate burdens in 2023–2024 also reported clear declines. Tuscany reduced admissions by 82%, from 66 to 12. Veneto fell from 116 to 27, a 77% reduction. Trentino-Alto Adige dropped by 79%, from 14 to 3. Umbria, which had reported 25 admissions in the previous season, nearly halved the number to 12. Piemonte reduced admissions from 13 to 7, a 46% decline. Sardinia reported a 78% reduction, from 9 admissions down to 2.

Smaller regions with limited admissions showed mixed patterns. Basilicata fell sharply from 11 to 3 cases, a 73% reduction. Marche recorded a fall from 10 to 2, equivalent to an 80% decrease. Calabria registered no cases in the 2024–2025 season compared to 3 previously, reflecting a complete elimination of PICU admissions. Abruzzo was an exception, with 4 admissions in both seasons, showing no change. Molise continued to report no admissions in either season. Data were not available for Liguria and Apulia, preventing direct comparison.

## Regional implementation of nirsevimab prophylaxis in Italian regions

**Fig. 1:**
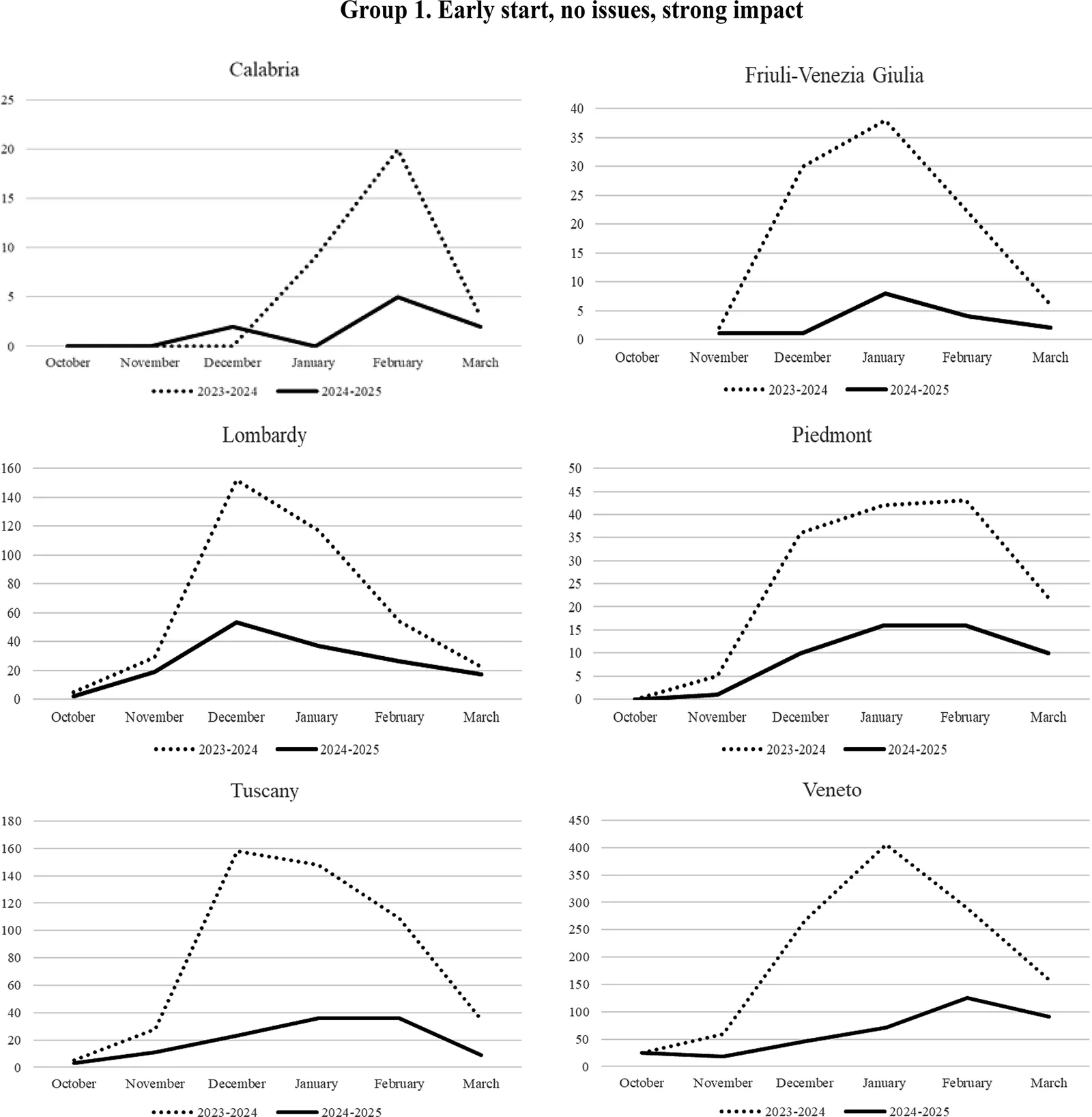
High-performing regions with early and smooth implementation, resulting in substantial reductions in hospital burden. On each graph, the admissions trends for RSV bronchiolitis during the epidemic season (from October to March) are shown by comparing the 2023–2024 and 2024–2025 two-year periods in the sentinel hospitals of the region

In Italy, the regional implementation of nirsevimab prophylaxis against RSV bronchiolitis showed clear variability in both timing and effectiveness, which can be grouped into three main performance clusters. We have classified the regions on the basis of their policies and of the disease outcome (Table 3). High-performing regions (group 1) included Calabria, Friuli-Venezia Giulia, Lombardy, Piedmont, Tuscany and Veneto which achieved the strongest outcomes, with more than a 60% reduction in hospital admissions for bronchiolitis (Fig. 1). These successes were largely due to an early start of prophylaxis, often in October 2024, combined with excellent coordination between neonatology units, pediatric services, and pharmacies. In these regions, coverage was broad, including infants born outside the main epidemic window, and no major drug supply constraints were reported. The result was not only a sharp reduction in hospitalizations but also a significant drop in pediatric intensive care unit (PICU) admissions. These regions represent a benchmark model for early activation, good planning, and seamless organizational integration.

**Table 3:** Groups of regions on the basis of their policies and performance outcomes

Group	Criteria	Regions	Summary
Group 1	Decrease in hospital admission for RSV bronchiolitis > 60%	Calabria, Friuli-Venezia Giulia, Lombardy, Piedmont, Tuscany, Veneto	High-performing regions with, in most cases, early and smooth implementation, no reported major issues, strongly reduction in PICU admissions
Group 2	Decrease in hospital admission for RSV bronchiolitis from 30% to 59%	Abruzzo, Apulia, Basilicata, Campania, Emilia-Romagna, Lazio, Liguria, Sardegna, Sicily, Trentino-Alto Adige	Decent impact on admissions, though not as consistent, prophylaxis started in November/January or limited immunization of out-of-season born, mild to moderate issues
Group 3	Decrease in hospital admission for RSV bronchiolitis < 25%	Umbria, Molise, Marche	Delayed activation and/or drug shortages likely limited the effectiveness of the intervention

**Fig. 2:**
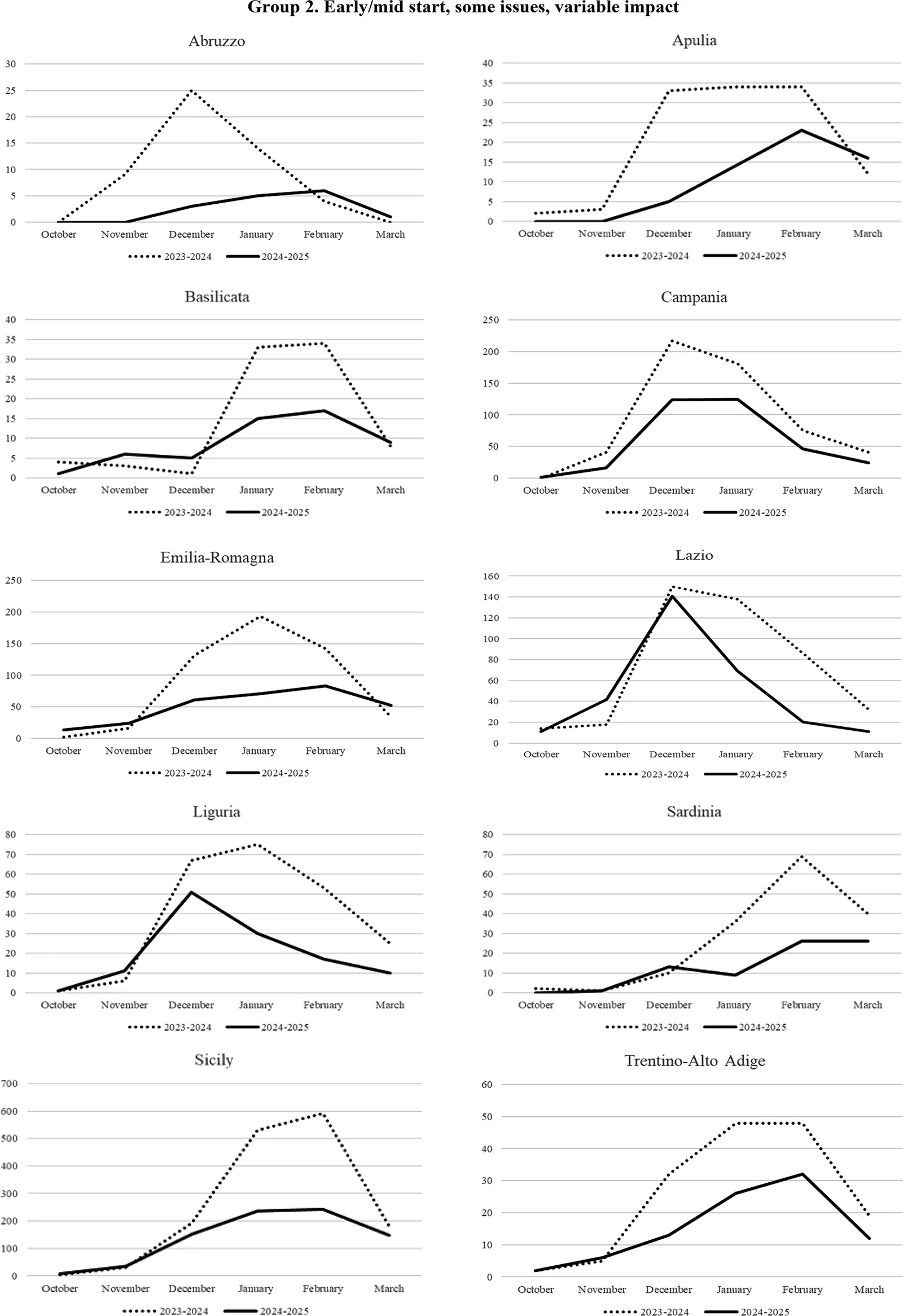
Regions with some operational challenges; decent impact on admissions, though not as consistent. On each graph, the admissions trends for RSV bronchiolitis during the epidemic season (from October to March) are shown by comparing the 2023–2024 and 2024–2025 two-year periods in the sentinel hospitals of the region

**Fig. 3:**
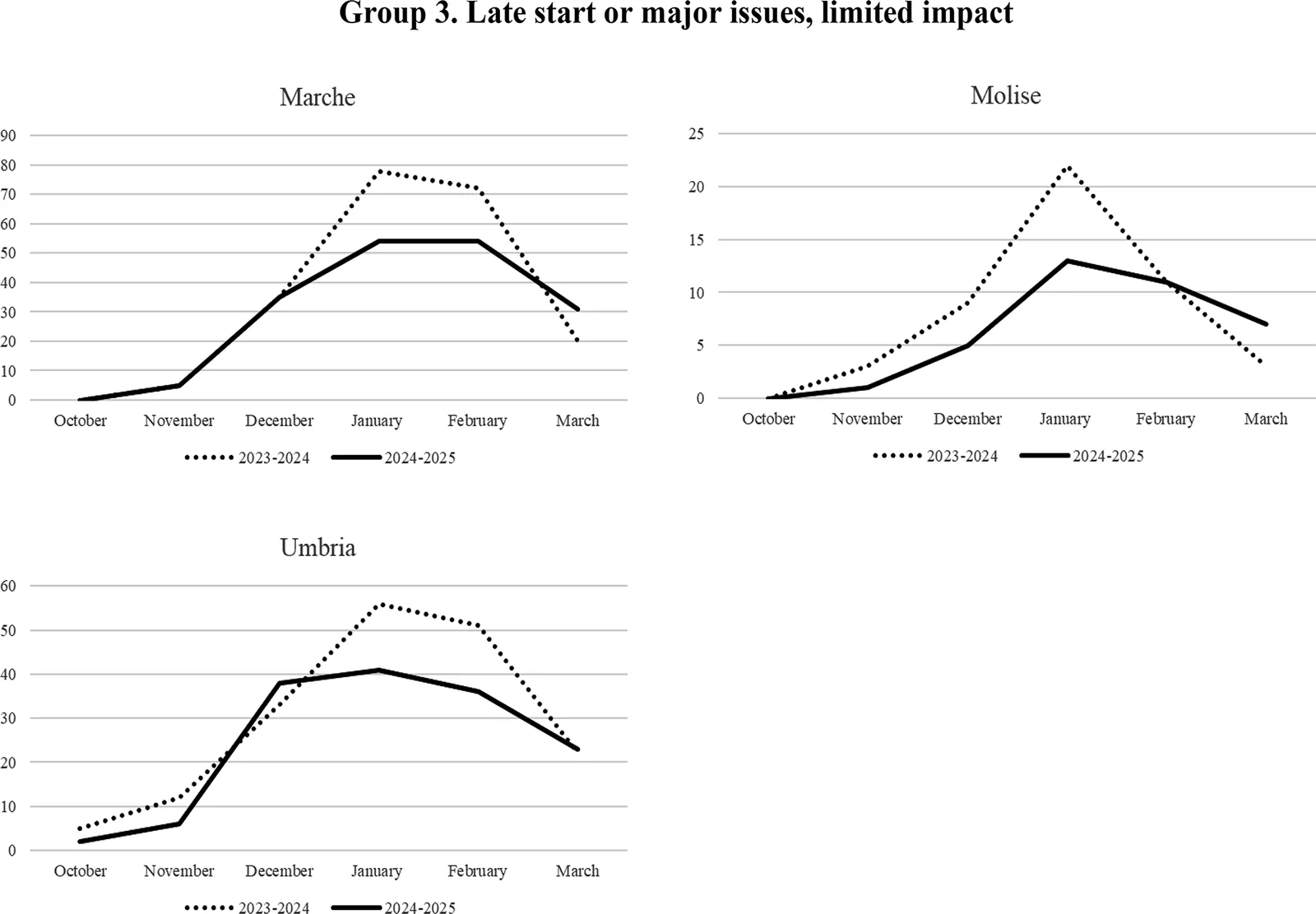
Regions with delayed activation and/or drug shortages likely limited the effectiveness of the intervention. On each graph are show the admissions trends for RSV bronchiolitis during the epidemic season (from October to March) by comparing 2023–2024 and 2024–2025 two-year period, in the sentinel hospitals of the region

Moderately effective regions (group 2), included Abruzzo, Apulia, Basilicata, Campania, Emilia-Romagna, Lazio, Liguria, Sardegna, Sicily, and Trentino-Alto Adige, saw reductions of 30–59% in RSV-related hospitalizations (Fig. 2). While the intervention was still effective, its impact was less consistent compared to Group 1. In several cases, prophylaxis started late (November or even December 2024), while in others coverage was more limited, particularly for infants born out of the epidemic peak season. Organizational barriers also played a role, including fragmented integration between hospitals and community healthcare, variable communication efforts towards families, and temporary but impactful drug supply shortages. The overall impact was still positive, but these regions would benefit from better planning and stronger logistical coordination to match the outcomes of the top-performing group. While other Regions showed delayed or ineffective implementation including (Group 3) Umbria, Molise, and Marche that experienced the least impact, with little or no improvement in hospitalization rates (reductions below 25%). Here, prophylaxis was generally launched only in December 2024 or January 2025, which coincided with or followed the peak of RSV infections, leaving many newborns unprotected (Fig. 3). Additional challenges included procurement delays and limited coordination across local health authorities. As a result, both hospital admissions and PICU admissions remained largely unchanged.

## Discussion

The implementation of nirsevimab across Italy revealed marked regional heterogeneity, mirroring patterns described in recent international studies. Northern and Central areas, such as Emilia-Romagna, Lombardy, Piedmont, Tuscany, Veneto, and Trentino-Alto Adige, launched campaigns earlier and with fewer logistical barriers, while Southern regions, including Calabria, Campania, Sardinia, and Molise, faced delays and organizational obstacles, often compounded by limited drug supply. These difficulties echo findings from Spain and France, where consistent drug availability and efficient planning were critical to achieving strong protective effects [19, 20]. In Catalonia and Andorra, systematic early immunization led to clear reductions in RSV hospitalizations, while in Paris, case-control analyses confirmed protection against bronchiolitis admissions. Additional evidence from French PICUs demonstrated an estimated 75.9% effectiveness in preventing severe RSV cases [21], and Andalusian data confirmed that timely prophylaxis reduced both hospitalizations and intensive care admissions [22]. Findings from Luxembourg similarly showed that population-wide prophylaxis substantially lowered RSV hospitalizations during the 2023–2024 season [23]. Together, these data highlight how early readiness, reliable supply, and coordinated execution consistently translate into measurable population benefits — an experience echoed in Italy, where regions with earlier activation were better able to avoid shortages and maintain smoother rollouts. In parallel, data on RSV-related hospitalizations in Italy showed a nationwide downward trend, with the sharpest declines in Friuli-Venezia Giulia, and more modest decreases in Marche, Molise, and Umbria. Some regions, such as Sicily, Campania, and Lombardy, continued to carry a high absolute burden, likely reflecting demographic factors and differences in preventive implementation. Nevertheless, reductions in pediatric intensive care unit (PICU) admissions were consistent across most regions, with the largest relative decreases observed in Friuli-Venezia Giulia, Tuscany, Calabria and Marche. Larger regions such as Lombardy, Emilia-Romagna, and Sicily still sustained the highest absolute numbers, but all demonstrated marked improvements compared to the prior season, suggesting a meaningful nationwide decline in severe RSV cases.

A combined analysis of prophylaxis rollout, pediatric hospital admissions, and PICU data stratified Italian regions into three performance groups. **Group 1** (Calabria, Friuli-Venezia Giulia, Lombardy, Piedmont, Tuscany and Veneto) represented high-performing areas, marked by early campaign activation, absence of major logistical barriers, and reductions in hospital burden often exceeding 60%. **Group 2** (Abruzzo, Apulia, Basilicata, Campania, Emilia-Romagna, Lazio, Liguria, Sardinia, Sicily, Trentino-Alto Adige) began later and faced operational hurdles such as intermittent shortages or bureaucratic delays, yet still achieved 30–60% reductions, albeit less consistently. **Group 3** (Marche, Molise, Umbria) experienced late activation and limited impact, with reductions generally below 25%.

The interpretation of these findings is limited by the lack of individual-level immunization coverage data, potential regional reporting bias, and reliance on administrative hospital records, which may lead to underestimation or misclassification of RSV bronchiolitis cases. In addition, substantial socioeconomic, demographic, and healthcare infrastructure differences across Italian regions represent important sources of heterogeneity that may influence hospitalization patterns and access to care. Marked regional variability exists in healthcare resources, particularly in the availability of pediatric intensive care units (PICUs). Italy has a limited number of PICUs nationwide, with a highly uneven regional distribution and several regions entirely lacking dedicated PICU facilities. This imbalance, which remains below European recommended standards for pediatric intensive care capacity, may substantially affect the observed regional distribution of severe RSV bronchiolitis. In regions with limited or absent PICU availability, critically ill infants may be transferred to referral centers in other regions or managed outside of PICUs, leading to potential under-ascertainment of severe cases locally. Conversely, regions with greater PICU capacity may appear to carry a disproportionate burden of severe disease, reflecting referral pathways and admission practices rather than true epidemiological differences.

Despite these limitations, the results of this study may offer useful insights for low- and middle-income countries, where constraints in healthcare infrastructure, limited access to intensive care, and heterogeneous implementation of preventive interventions are common. The observed reductions in RSV-related hospitalizations following the introduction of passive immunization strategies, even within a fragmented healthcare system, suggest that population-level benefits may be achievable in settings with limited resources. However, direct generalization to low-income countries should be approached with caution, as differences in baseline RSV epidemiology, healthcare access, referral systems, socioeconomic conditions, and data collection capacity may substantially modify both the effectiveness and the measurable impact of prophylactic interventions.

Taken together, these findings underscore the importance of early planning, reliable supply chains, and organizational efficiency in maximizing the impact of nirsevimab prophylaxis. Regions that were able to start early and operate smoothly achieved the most substantial reductions in both pediatric and intensive care admissions. Conversely, delayed or troubled rollouts yielded only modest benefits, reinforcing the time-sensitive nature of RSV prevention.

## Data Availability

the datasets analysed during the current study are available from the corresponding author on reasonable request.

## Declarations

This study was conducted through the analysis of hospital discharge forms (SDOs) for bronchiolitis and therefore did not require access to individual patient data. The study is exclusively based on administrative datasets.

## References

[1] Azzari C, Baraldi E, Bonanni P, Bozzola E, Coscia A, Lanari M, et al. Epidemiology and prevention of respiratory syncytial virus infections in children in Italy. Ital J Pediatr [Internet]. BioMed Central Ltd; 2021 [cited 2025 Jul 10];47. 10.1186/S13052-021-01148-8. 10.1186/s13052-021-01148-8 PMC8487331 34600591

[2] Muñoz-Quiles C, López-Lacort M, Díez-Domingo J, Orrico-Sánchez A, Bronchiolitis. Regardless of Its Etiology and Severity, Is Associated With Increased Risk of Asthma: A Population-Based Study. Volume 228. Oxford University Press; 2023. pp. 840–50. [cited 2025 Jul 10];. 10.1093/INFDIS/JIAD093. Journal of Infectious Diseases [Internet]. 10.1093/infdis/jiad093 PMC10547461 37015894

[3] Madhi SA, Ceballos A, Cousin L, Domachowske JB, Langley JM, Lu E, et al. Population attributable risk of wheeze in 2–<6-Year-old Children, following a respiratory syncytial virus lower respiratory tract infection in the first 2 years of Life. Pediatric infectious disease journal. Lippincott Williams Wilkins. 2025;44:379–86. 10.1097/INF.0000000000004447. 10.1097/INF.0000000000004447 38985986

[4] van Wijhe M, Johannesen CK, Simonsen L, Jørgensen IM, Fischer TK. A Retrospective Cohort Study on Infant Respiratory Tract Infection Hospitalizations and Recurrent Wheeze and Asthma Risk: Impact of Respiratory Syncytial Virus. Volume 226. Oxford University Press; 2022. pp. S55–62. [cited 2025 Jul 10];. 10.1093/INFDIS/JIAC141. Journal of Infectious Diseases [Internet]. 10.1093/infdis/jiac141 35426942

[5] Manti S, Staiano A, Orfeo L, Midulla F, Marseglia GL, Ghizzi C, et al. UPDATE – 2022 Italian guidelines on the management of bronchiolitis in infants. Ital J Pediatr [Internet] Ital J Pediatr. 2023. 10.1186/S13052-022-01392-6. [cited 2025 Oct 17];49. 36765418 10.1186/s13052-022-01392-6 PMC9912214

[6] Camporesi A, Morello R, Ferro V, Pierantoni L, Rocca A, Lanari M, et al. Epidemiology, microbiology and severity of bronchiolitis in the first Post-Lockdown cold season in three different geographical areas in italy: A Prospective, observational study. Children. 2022;9:491. 10.3390/children9040491. 35455535 10.3390/children9040491 PMC9024462

[7] Zhu T, Zhang C, Yu L, Chen J, Qiu H, Lyu W, et al. The preventive effect of vaccine prophylaxis on severe respiratory syncytial virus infection: A meta-analysis. Virol Sin. 2015;30:371–8. 10.1007/s12250-015-3630-3. 26511990 10.1007/s12250-015-3630-3 PMC8200922

[8] Alderton G. Progress in RSV prevention. Science (1979). 2021;372:698.9–700. 10.1126/science.372.6543.698-i

[9] Robinson RF, Nahata MC. Respiratory syncytial virus (RSV) immune Globulin and Palivizumab for prevention of RSV infection. Am J Health-System Pharm. 2000;57:259–64. 10.1093/ajhp/57.3.259. 10.1093/ajhp/57.3.259 10674778

[10] Bollani L, Baraldi E, Chirico G, Dotta A, Lanari M, Del Vecchio A, et al. Revised recommendations concerning Palivizumab prophylaxis for respiratory syncytial virus (RSV). Ital J Pediatr. 2015;41:97. 10.1186/s13052-015-0203-x. 26670908 10.1186/s13052-015-0203-x PMC4681171

[11] Garegnani L, Roson Rodriguez P, Escobar Liquitay CM, Esteban I, Franco JVA. Palivizumab for preventing severe respiratory syncytial virus (RSV) infection in children. Cochrane Database Syst Rev [Internet] Cochrane Database Syst Rev. 2025. 10.1002/14651858.CD013757.PUB3. [cited 2025 Sep 18];7. 40698576 10.1002/14651858.CD013757.pub3 PMC12284916

[12] Griffin MP, Yuan Y, Takas T, Domachowske JB, Madhi SA, Manzoni P, et al. Single-Dose nirsevimab for prevention of RSV in preterm infants. New Engl J Med Mass Med Soc. 2020;383:415–25. 10.1056/nejmoa1913556. 10.1056/NEJMoa1913556 32726528

[13] Hammitt LL, Dagan R, Yuan Y, Baca Cots M, Bosheva M, Madhi SA, et al. Nirsevimab for prevention of RSV in healthy Late-Preterm and term infants. N Engl J Med [Internet] N Engl J Med. 2022;386:837–46. 10.1056/NEJMOA2110275. [cited 2025 Jul 10];. 35235726 10.1056/NEJMoa2110275

[14] Manti S, Baraldi E. Learn from international recommendations and experiences of countries that have successfully implemented monoclonal antibody prophylaxis for prevention of RSV infection. Ital J Pediatr BioMed Cent Ltd. 2025;51. 10.1186/s13052-025-01844-9. 10.1186/s13052-025-01844-9 PMC11796128 39905524

[15] Orsi A, Scarpaleggia M, Baldo V, Barbone F, Chironna M, Giuffrida S, et al. First real-world data on universal respiratory syncytial virus prophylaxis with nirsevimab in infants. J Prev Med Hyg [Internet] J Prev Med Hyg. 2024;65:E172–87. 10.15167/2421-4248/JPMH2024.65.2.3329. [cited 2025 Sep 18];. 39430977 10.15167/2421-4248/jpmh2024.65.2.3329 PMC11487721

[16] Attaianese F, Trapani S, Agostiniani R, Ambrosino N, Bertolucci G, Biasci P, et al. Effectiveness of a targeted infant RSV immunization strategy (2024–2025): A multicenter matched case-control study in a high-surveillance setting. Journal of Infection [Internet]. W.B. Saunders Ltd; 2025 [cited 2025 Sep 18];91. 10.1016/j.jinf.2025.106600 10.1016/j.jinf.2025.106600 40848988

[17] Consolati A, Farinelli M, Serravalle P, Rollandin C, Apprato L, Esposito S, et al. Safety and efficacy of nirsevimab in a universal prevention program of respiratory syncytial virus bronchiolitis in newborns and infants in the first year of life in the Valle d’aosta Region, Italy, in the 2023–2024 epidemic season. Vaccines (Basel). 2024;12:549. 10.3390/vaccines12050549. 38793800 10.3390/vaccines12050549 PMC11125727

[18] Lastrucci V, Pacifici M, Alderotti G, Puglia M, Berti E, Barbati F, et al. The impact of nirsevimab prophylaxis on RSV hospitalizations: a real-world cost-benefit analysis in Tuscany, Italy. Front Public Health. 2025;13. 10.3389/fpubh.2025.1604331. 10.3389/fpubh.2025.1604331 PMC12267333 40678647

[19] Agüera M, Soler-Garcia A, Alejandre C, Moussalam‐Merino S, Sala‐Castellví P, Pons G, et al. Nirsevimab immunization’s real‐world effectiveness in preventing severe bronchiolitis: A test‐negative case–control study. Pediatr Allergy Immunol. 2024;35. 10.1111/pai.14175. 10.1111/pai.14175 38899631

[20] Carbajal R, Boelle P-Y, Pham A, Chazette Y, Schellenberger M, Weil C, et al. Real-world effectiveness of nirsevimab immunisation against bronchiolitis in infants: a case–control study in Paris, France. Lancet Child Adolesc Health. 2024;8:730–9. 10.1016/S2352-4642(24)00171-8. 39208832 10.1016/S2352-4642(24)00171-8

[21] Paireau J, Durand C, Raimbault S, Cazaubon J, Mortamet G, Viriot D, et al. Nirsevimab effectiveness against cases of respiratory syncytial virus bronchiolitis hospitalised in paediatric intensive care units in France, September 2023–January 2024. Influenza Other Respir Viruses. 2024;18. 10.1111/irv.13311. 10.1111/irv.13311 PMC11154801 38840301

[22] Moreno-Pérez D, Korobova A, Croche-Santander F, de B, Cordón-Martínez A, Díaz-Morales O, Martínez-Campos L, et al. Nirsevimab prophylaxis for reduction of respiratory syncytial virus complications in hospitalised infants: the Multi-Centre study during the 2023–2024 season in Andalusia, Spain (NIRSEGRAND). Vaccines (Basel). 2025;13:175. 10.3390/vaccines13020175. 40006722 10.3390/vaccines13020175 PMC11861336

[23] Ernst C, Bejko D, Gaasch L, Hannelas E, Kahn I, Pierron C, et al. Impact of nirsevimab prophylaxis on paediatric respiratory syncytial virus (RSV)-related hospitalisations during the initial 2023/24 season in Luxembourg. Eurosurveillance. 2024;29. 10.2807/1560-7917.ES.2024.29.4.2400033. 10.2807/1560-7917.ES.2024.29.4.2400033 PMC10986653 38275017

